# Identification of apoptosis-immune-related gene signature and construction of diagnostic model for sepsis based on single-cell sequencing and bulk transcriptome analysis

**DOI:** 10.3389/fgene.2024.1389630

**Published:** 2024-06-04

**Authors:** Zhongyi Sun, Yanan Hu, Jiachen Qu, Qiuyue Zhao, Han Gao, Zhiyong Peng

**Affiliations:** ^1^ Department of Critical Care Medicine, Zhongnan Hospital of Wuhan University, Wuhan, China; ^2^ Clinical Research Center of Hubei Critical Care Medicine, Wuhan, China; ^3^ Department of Pulmonary Medicine, Zhongnan Hospital of Wuhan University, Wuhan, China

**Keywords:** apoptosis, bioinformatics, function analysis, gene signature, immune landscape, sepsis

## Abstract

**Introduction:**

Sepsis leads to multi-organ dysfunction due to disorders of the host response to infections, which makes diagnosis and prognosis challenging. Apoptosis, a classic programmed cell death, contributes to the pathogenesis of various diseases. However, there is much uncertainty about its mechanism in sepsis.

**Methods:**

Three sepsis gene expression profiles (GSE65682, GSE13904, and GSE26378) were downloaded from the Gene Expression Omnibus database. Apoptosis-related genes were obtained from the Kyoto Encyclopedia of Genes and Genomes Pathway database. We utilized LASSO regression and SVM-RFE algorithms to identify characteristic genes associated with sepsis. CIBERSORT and single cell sequencing analysis were employed to explore the potential relationship between hub genes and immune cell infiltration. The diagnostic capability of hub genes was validated across multiple external datasets. Subsequently, the animal sepsis model was established to assess the expression levels of hub genes in distinct target organs through RT-qPCR and Immunohistochemistry analysis.

**Results:**

We identified 11 apoptosis-related genes as characteristic diagnostic markers for sepsis: *CASP8*, *VDAC2*, *CHMP1A*, *CHMP5*, *FASLG*, *IFNAR1*, *JAK1*, *JAK3*, *STAT4*, *IRF9*, and *BCL2*. Subsequently, a prognostic model was constructed using LASSO regression with *BCL2*, *FASLG*, *IRF9* and *JAK3* identified as hub genes. Apoptosis-related genes were closely associated with the immune response during the sepsis process. Furthermore, in the validation datasets, aside from *IRF9*, other hub genes demonstrated similar expression patterns and diagnostic abilities as observed in GSE65682 dataset. In the mouse model, the expression differences of hub genes between sepsis and control group revealed the potential impacts on sepsis-induced organ injury.

**Conclusion:**

The current findings indicated the participant of apoptosis in sepsis, and apoptosis-related differentially expressed genes could be used for diagnosis biomarkers. *BCL2*, *FASLG*, *IRF9* and *JAK3* might be key regulatory genes affecting apoptosis in sepsis. Our findings provided a novel aspect for further exploration of the pathological mechanisms in sepsis.

## 1 Introduction

Sepsis is one of the most important healthcare problems evoking ongoing researches about pathogenesis, diagnosis, therapy, and prognosis (Kumar, 2018). Several elements were certified to be involved in the sepsis mechanism, including pathogenic microorganisms, systemic inflammatory response, abnormal energy metabolism, immunosuppression, and multiple targeting organs (Hawiger et al., 2015; Stone, 2017; Huang et al., 2019). According to “Sepsis-3” consensus definition, sepsis was defined as a life-threatening organ dysfunction caused by dysregulated host response to infection (Singer et al., 2016). Over 45 million people suffer from sepsis worldwide, with a high fatality of 16.7%–33.3% each year (Rhee et al., 2017; Fleischmann-Struzek et al., 2020; Rudd et al., 2020). Although remarkable progress has been made in anti-infective treatments, fluid resuscitation, multiple organ support, or other medical technologies, initial uncontrolled host response to infection still leads to the malignant development of sepsis and adverse clinical outcomes (Rello et al., 2017; Evans et al., 2021).

In recent decades, the effects of cell death have emerged as the breakthrough to establish more thorough theories of sepsis, which can be broadly divided into programmed cell death (PCD) and accidental cell death (ACD) (Hotchkiss et al., 2009; D'Arcy, 2019). ACD manifests as a biologically uncontrolled process with representation by necrosis. In contrast, PCD is an active suicide of cells conducted by a cascade of signal reactions and molecular effects facing excessive cellular stress, typically represented by apoptosis (Shi et al., 2021). During apoptosis, the activation of executioner caspases contributed to clearance of intracellular components in an accelerated manner and ultimate phagocytosis of dying cells (Chipuk and Green, 2005). In sepsis, there exhibits a substantial excessive apoptosis of tissue cells triggered by the intense inflammatory response, meanwhile, the apoptosis of cells of the innate and adaptive immune system might impact the body ability to combat infections (Hotchkiss and Nicholson, 2006; Henson and Bratton, 2013). However, the role of apoptosis is not solely adverse in sepsis. Researchers have found that moderate apoptosis aided in clearing infected cells and limiting the spread of pathogens in the early stage of sepsis (Lang and Matute-Bello, 2009). By means of intervening in apoptotic signaling pathways, it is possible to regulate the extent of cell apoptosis and mitigate organ damage, thereby achieving positive therapeutic effects on sepsis (Xiao et al., 2023). This requires a more comprehensive consideration of the overall balance of immune system to avoid adverse consequences. In summary, in-depth researches into the molecular mechanisms of apoptosis facilitate the improvement of diagnosis and clinical decisions for sepsis. There is still a need for more explorations to address the questions of how to balance the promotion and inhibition of apoptosis, as well as determining optimal intervention at different stages (Delsesto et al., 2011).

Therefore, in the present study, we carried out a systematic bioinformatic analysis based on the Gene Expression Omnibus (GEO) database to delineate apoptosis-related gene signatures for the diagnosis and/or prognosis of sepsis, further, we evaluate the potential relationship between apoptosis and immune microenvironment in sepsis.

## 2 Methods

### 2.1 Data acquisition and processing

The GSE65682, GSE13904, and GSE26378 datasets were retrieved from the Gene Expression Omnibus (GEO) database (www.ncbi.nlm.nih.gov/geo/). The GSE65682 dataset contained 802 blood samples: 760 sepsis and 42 healthy volunteers based on the GPL3667 platform. The GSE13904 and GSE26378, covering pediatric sepsis gene expression data, were used for external validation. The former dataset included 18 healthy children, 52 sepsis subjects, and 106 subjects with septic shock based on the GPL570 platform. The latter comprised 21 normal children and 82 sepsis cases, also based on the GPL570 platform (Table 1). The profiles of apoptosis-related genes were obtained from the Kyoto Encyclopedia of Genes and Genomes (KEGG) pathway database (https://www.genome.jp/entry/map04210). Then, data normalization and variation analysis were conducted using the “limma” R package. Heatmap was generated under *p* < 0.05 using the “pheatmap” R package. All data were collected from public databases; hence, ethics approval and informed consent were not required. The overview of the workflow steps is shown in Supplementary Figure S1.

**TABLE 1:** Information of the microarray datasets.

Dataset	Platform	Samples	Control	Sepsis
GSE65682	GPL13667 (HG-U219) Affymetrix Human Genome U219 Array	802	42	760
GSE26378	GPL570 (HG-U133_Plus_2) Affymetrix Human Genome U133 Plus 2.0 Array	103	21	82
GSE13904^a^	GPL570 (HG-U133_Plus_2) Affymetrix Human Genome U133 Plus 2.0 Array	227	18	158
GSE167363	GPL24676 Illumina NovaSeq 6000 (*Homo sapiens*)	12	2	10

^a^ The GSE13904 contained several samples which is independent of this research, only the healthy and sepsis cases were extracted for further study.

### 2.2 Functional enrichment analysis of apoptosis-related genes

To study the molecular functions (MFs), biological processes (BPs), and cellular components (CCs) of apoptosis-related genes, “clusterProfiler,” “org.Hs.e.g.,.db,” and “enrichplot” R packages were used for Gene Ontology (GO) annotation. KEGG pathway enrichment analysis was also conducted. The threshold for each analysis was set at *p* < 0.05.

### 2.3 Selection of optimal apoptosis-related gene biomarkers for sepsis

We applied least absolute shrinkage and selection operator (LASSO) regression and support vector machine-recursive feature elimination (SVM-RFE) model for disease-specific gene selection. The optimal λ selected returned the minimum cross-validation error under 10-fold cross-validation in the LASSO model. The SVM-RFE model was executed using the “SVM” R package. Each feature’s score was sorted after removing the minimum value and iterations were sustainably performed until the best features were selected. Accordingly, differentially expressed genes (DEGs) for sepsis were identified by overlapping both results. Moreover, the diagnostic ability of DEGs was assessed using receiver operating characteristic (ROC) curves with the “pROC” R package. The volcano plot and heatmap presented the detailed expression of DEGs with “ggplot” and “pheatmap” R package.

### 2.4 Verification and survival analysis of hub genes

The univariate Cox and LASSO regressions with Candidate DEGs were conducted using the “glmnet” R package to construct a prognostic signature. Then, the “timeROC” R package was employed to perform 1-, 2- and 3-week ROC analyses, and results were quantified by the area under curve (AUC). Meanwhile, the Kaplan-Meier survival analysis was conducted using ‘‘survival” and ‘‘survminer” R packages. Using the formula Risk score = (each gene’s expression×corresponding coefficient), septic patients were classified into the high-risk group (risk score > median risk score) and low-risk group (risk score ≤ median risk score). The entire dataset was divided into train and test set, keeping a ratio of about 1:1. These analyses were carried out on the whole dataset, train dataset, and test dataset, respectively.

### 2.5 Nomogram development

The nomogram was built using the multivariate Cox regression with the “survival” and “rms” R packages. The concordance index (C-index) was applied to assess the discriminatory performance of hub genes. The calibration curve was applied for nomogram calibration. This algorithm can be an efficient graphical description in which each risk factor responds to the risk of mortality at 1-, 2-, and 3- week for an individual patient.

### 2.6 Single gene set enrichment analysis (GSEA) and single gene set variation analysis (GSVA)

To study transcriptome differences and personalized analysis, GSEA was carried out using the “GSEABase” R package. According to the log_2_ [Fold Change (FC)] value of the differential analysis, genes were ranked from high to bottom to be defined as the tested gene set. The KEGG pathway set was applied to conduct pathway-level analysis of each hub gene using the “GSVA” R package, in which the relative pathway activity represented as the t-value. Up- and downregulated pathways were determined using the “limma” R package.

### 2.7 Immune infiltration analysis

CIBERSORT is a classic method to quantify the relative abundance of various cell composition from enormous gene expression profile (Chen et al., 2018). By means of CIBERSORT in the GSE65682, the proportion of 22 types of infiltrating immune cells was analyzed between sepsis and control groups, further, the correlation degree between immune cells and hub genes was calculated using Pearson correlation. The results were shown in visual diagrams via “ggplot” R language.

### 2.8 Analysis of single cell RNA sequencing (scRNA-seq)

Based on GEO database, GSE167363 raw data was downloaded. Single cells were extracted under the criteria: nFeature_RNA>500, percent. mt<20%, percent. HB < 1, nCount_RNA>1000. The doublet and non-viable cells were removed by “DoubletFinder” R language and Scrublet algorithm. Normalization, scaling, clustering of cells was implemented by “Seurat” R package, which generated dominant immune cell types. The results were distinguished using t-distributed stochastic neighbor embedding (tSNE). The average gene expression levels in each identified cell type were estimated using “Seurat” R package. The Variation analyses were conducted using the “limma” R package.

### 2.9 Validation of expression of hub genes in external datasets

For high quality evidence in external validation, we not only employed GSE13904, GSE26378 form GEO database, also harvested 158 adult samples from ZhongNan hospital, Wuhan University, composed of 15 healthy volunteers and 143 septic patients. All patients were adequately informed, and the study received approval from the Ethics Committee of ZhongNan Hospital, Wuhan University (2017004). The demographic and clinical characteristics of recruited samples were summarized in Supplementary Table S1. The peripheral blood mononuclear cells (PBMCs) were collected from patients on the first day after the diagnosis of sepsis using leucocyte cell separation medium kit (TBD Science, Tianjin, China), further, the total RNA was extracted. Based on thorough evaluation of its integrity and quality, we selected RNA with the RNA Integrity Number (RIN)≥7.0 and 28S/18S ratio>1.0 for library preparation and mRNA transcriptome sequencing. Subsequently, the library was constructed through the TruSeq Stranded mRNA Library Prep Kit (Illumina), and sequencing was performed using NovaSeq 6000 (Illumina). Regarding the external datasets validation, following the calculation of expression level of hub genes using RSEM (Li and Dewey, 2011) in two separate groups, differential expression analysis was performed using the “DESeq2” R package with *p* < 0.05, and the diagnostic value of hub genes was presented with the fitting ROC curves. Plus, the volcano plot and heatmap revealed the intuitive expression of hub genes. Besides, we explored the prognostic significance of hub genes shown in 1-, 2- and 3-week ROC analyses of Zhongnan Hospital dataset.

### 2.10 Animals and grouping

C57BL/6 mice (males and 8 weeks old) were housed under a standard raising condition. Ten mice were randomly divided into control and sepsis groups with five in each group. The sepsis model was established using the cecal ligation and puncture (CLP) method according to guidelines (Rittirsch et al., 2008). After anesthetizing mice with pentobarbital sodium solution (0.01 mg/g) by intraperitoneal injection, the caecum was exposed and ligatured during laparotomy. The major step was the caecal wall puncture with a 20-gauge needle and gently squeezing the stool (length: 2 mm). The surgery ended with abdomen closure layer by layer. The heating pad and normal saline injection were used for better post-operation resuscitation. In the control group, the abdomen was only opened and closed without extra operation. After 12 h, blood samples were collected and centrifuged to obtain serum. Subsequently, the mice were humanely euthanized with pentobarbital sodium (0.1 mg/g), then, the heart, lung, liver, and kidney tissues were harvested for further experiments. The Animal Care and Use Committee of the Zhongnan Hospital of Wuhan University approved the animal experiment.

### 2.11 Enzyme linked immunosorbent assay (ELISA)

Following the extraction of mice serum in control and CLP groups, the creatinine (Cr) level was determined by Creatinine Assay Kit (Sigma-Aldrich, Shanghai, China) and blood urea nitrogen (BUN) level were determined by BUN Quick Test Strips (Sigma-Aldrich, Shanghai, China) according to the manufacturer’s instructions.

### 2.12 Real-time quantitative polymerase chain reaction (RT-qPCR) validation

Following the samples harvested from mice models, total RNA was extracted using the FastPure Plant Total RNA Isolation Kit (Vazyme, Nanjing, China). Then, cDNA was synthesized using the Hifair^®^ first Strand cDNA Synthesis SuperMix (Yeasen Biotechnology, Shanghai, China) with the following procedure: 25°C/5 min, 42°C/30 min, and 85°C/5 min. Next, we performed qPCR using ChamQ SYBR qPCR Master Mix (Vazyme Biotech, Nanjing, China). The amplification process was implemented in the Roche LightCycler^®^96 Real-Time PCR Detection System (Hoffmann-La Roche Ltd., Shanghai, China). The primer pairs used were presented in Table 2. With GAPDH serving as reference gene, the expression levels of hub genes in serum and target organs (heart, lung, liver and kidney) were analyzed using the 2^−ΔΔCT^ method (Livak and Schmittgen, 2001). In general, GAPDH had an identical presentation despite distinct treatments in two groups, the relative mRNA abundance of each gene was estimated as the fold change of its expression level in CLP groups over that in control groups.

**TABLE 2:** Primer sequences used for RT-qPCR.

Gene	Forward sequence (5′-3′)	Reverse sequence (5′-3′)
BCL2	GGA​TTG​TGG​CCT​TCT​TTG​AGT​TC	CTT​CAG​AGA​CAG​CCA​GGA​GAA​AT
FASLG	GGC​TCT​GGT​TGG​AAT​GGG​ATT​AG	AGA​GAT​CAG​AGC​GGT​TCC​ATA​TG
IRF9	GAG​CTC​TTC​AAG​ACC​ACC​TAC​TT	TAA​CAG​GAA​CAA​GGC​AGC​TTT​CT
JAK3	CGC​AGG​ACT​ATG​ACA​GCT​TTC​TT	GTC​TAC​TCG​CAG​CCC​AGA​ATT​C
GAPDH	GGC​ATT​GTG​GAA​GGG​CTC​AT	AGA​TCC​ACG​ACG​GAC​ACA​TT

### 2.13 Immunohistochemistry (IHC) analysis

As described above, the heart, lung, liver, and kidney tissues were acquired and made into paraffin tissue slides. Sections were dewaxed through xylene, ethanol, and distilled water. Sodium Citrate Antigen Retrieval Solution (Solarbio Science and Technology, Beijing, China) was used to recover antigens. After 30-min blocking of Bovine Serum Albumin (Solarbio Science and Technology, Beijing, China), primary antibodies were added to sections and incubated overnight at 4°C. The source of primary antibodies was listed as following: anti-BCL2 antibody: Proteintech, Cat No. 68103 at 1/800 dilution; anti-FASLG antibody: Proteintech, Cat No. 60196 at 1/200 dilution; anti-IRF9 antibody: Proteintech, Cat No. 14167 at 1/500 dilution; anti-JAK3 antibody: Proteintech, Cat No. 80331 at 1/500 dilution. After that step, the sections were washed three times in Phosphate Buffered Solution (PBS) and incubated with secondary antibody at room temperature for 20 min. The final chromogenic reaction was conducted using the DAB Substrate kit (Solarbio Science and Technology, Beijing, China), and the sections were dehydrated and sealed. The target proteins were stained in brown and further histological analysis was completed by an experienced pathologist using a light microscope in a blinded method. The percentage of positive expression of target proteins was independently assessed in four kinds of organs. All the images were shown at 30* magnification.

### 2.14 Apoptosis detection

Apoptosis of heart, lung, liver and kidney were evaluated through the terminal deoxynucleotidyl transferase-mediated fluorescein-dUTP nick-end labeling (TUNEL) technique using the Click-iT™ Plus TUNEL Assay Kit (ThermoFisher Scientific, Shanghai, China). The results were observed under Nikon Eclipse 50i Fluorescence Upright Microscope (Tokyo, Japan).

### 2.15 Statistical analysis

Statistical analyses were performed using R software. Comparisons between two groups were assessed with the Student’s t-test. Unless stated otherwise, statistical tests were bilateral, with *p* < 0.05 as significant. The Kaplan-Meier method was applied to compare the overall survival (OS) time. The RT-qPCR results were interpreted using GraphPad Prism 8. The IHC and TUNEL images were rendered with Adobe Photoshop 2022.

## 3 Results

### 3.1 Identification and functional analysis of apoptosis-related genes

**FIGURE 1:**
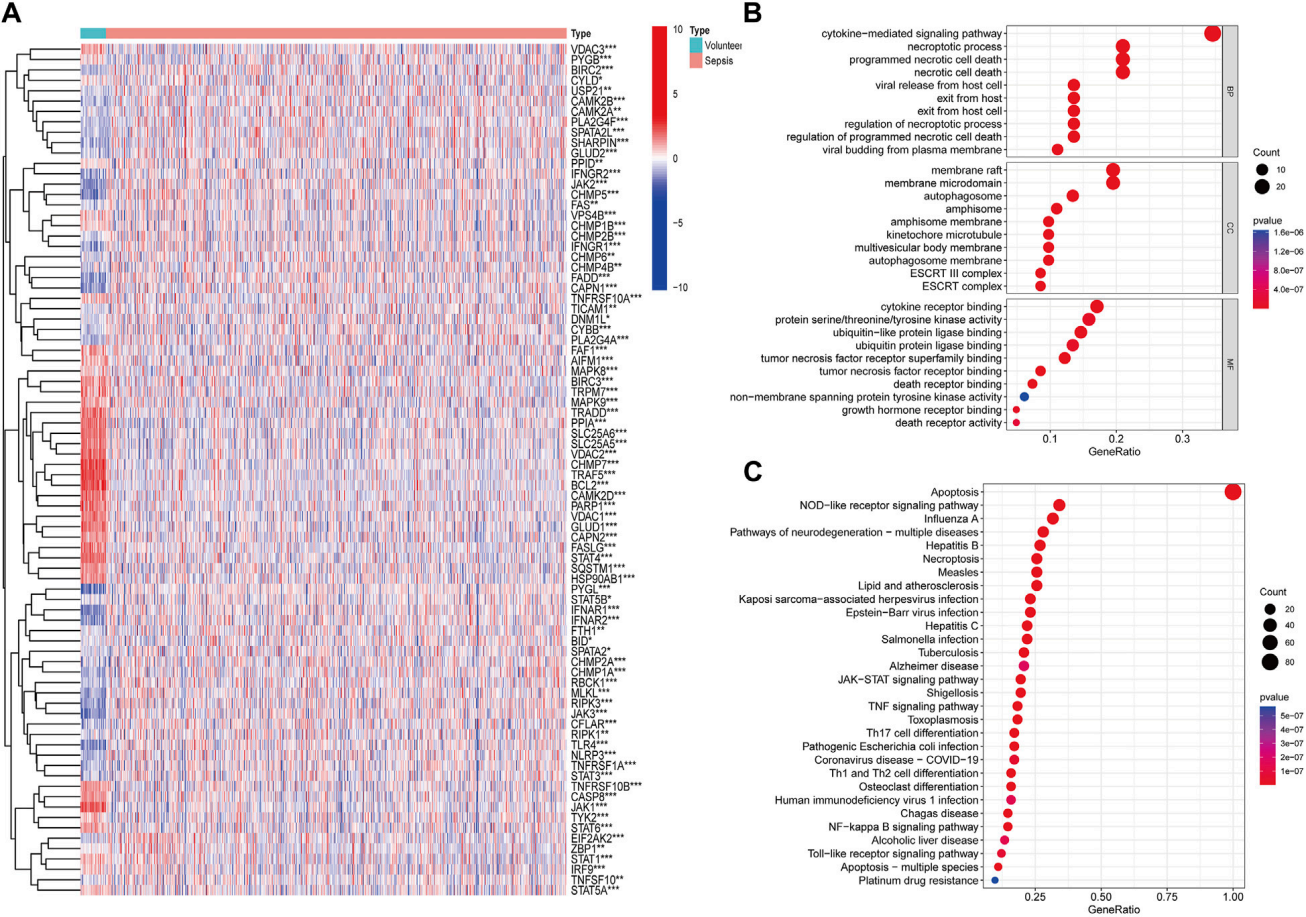
Identification and function analysis of apoptosis-related genes. **(A)** Heatmap of apoptosis-related genes identified in GSE65682 (*p < 0.05, **p < 0.01, ***p < 0.001). **(B)** Gene Ontology (GO) annotation of apoptosis-related genes. **(C)** Kyoto Encyclopedia of Genes and Genomes (KEGG) pathway enrichment analysis of apoptosis-related genes.

With access to the apoptosis-related genes, 82 genes were differently expressed in sepsis in the GSE65682 cohort (Figure 1A). Then, we conducted GO functional and KEGG pathway enrichment analysis to screen predominant signaling pathways. The top 10 significantly enriched terms in BPs, CCs, and MFs are presented in Figure 1B, and the top 30 KEGG pathways in Figure 1C. The preliminary results suggested that apoptosis-related pathways were enriched and upregulated in sepsis, and apoptosis-related genes were linked to apoptosis pathway, necroptosis pathway, NOD-like receptor signaling pathway, JAK-STAT signaling pathway, NF-kappa B signaling pathway, et al., reflecting the strong relationship between apoptosis and sepsis.

### 3.2 Eleven DEGs were identified as diagnostic genes for sepsis

**FIGURE 2:**
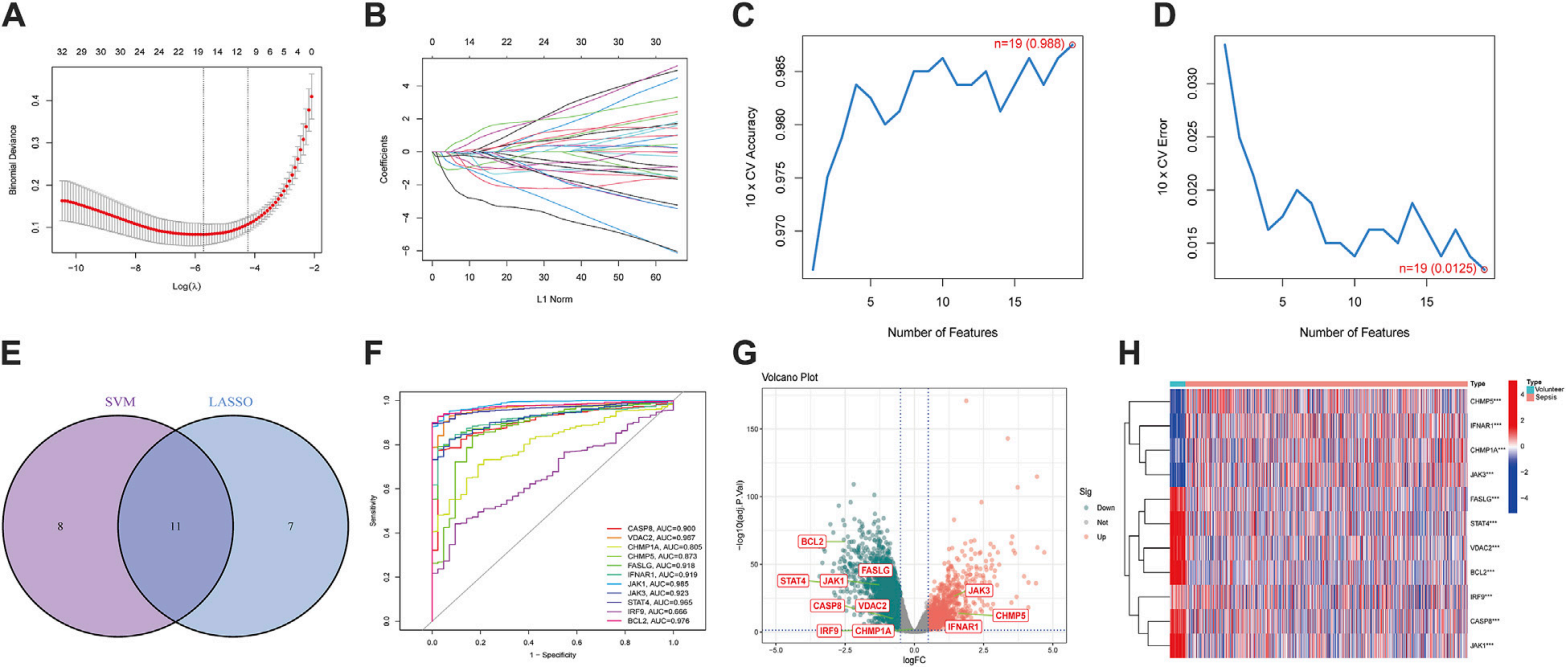
Screening of apoptosis-related differentially expressed genes (DEGs) and exploration of diagnostic ability. **(A,B)** 18 differentially expressed features were selected by LASSO regression algorithm. **(C,D)** 19 differentially expressed features were regarded as the optimal genes by SVM-RFE algorithm. **(E)** DEGs were acquired from the intersection of LASSO and SVM-RFE. **(F)** The diagnostic ROC curves of DEGs. **(G)** The volcano plot of DEGs. **(H)** Heatmap of DEGs. (*p < 0.05, **p < 0.01, ***p < 0.001).

We performed two machine-learning algorithms: LASSO and SVM-RFE. The LASSO regression showed 18 apoptosis-related features (Figures 2A, B), the SVM-RFE algorithm filtered 19 genes as the optimal combination of feature genes (maximal accuracy = 0.988, minimal root mean square error = 0.0125) (Figures 2C, D). After intersecting both results, 11 DEGs were selected: *CASP8, VDAC2, CHMP1A, CHMP5, FASLG, IFNAR1, JAK1, JAK3, STAT4, IRF9,* and *BCL2* (Figure 2E). Then, we established ROC curves indicated the AUC of nine genes were >0.85 (Figure 2F). The volcano plot and heatmap indicated that *JAK3*, *CHMP5* and *IFNAR1* were upregulated while *CASP8, VDAC2, FASLG, JAK1, STAT4* and *BCL2* were downregulated in sepsis (Figures 2G, H). These results showed the superiority of filtered DEGs in distinguishing sepsis from healthy cases.

### 3.3 Establishment of prognostic signature based on DEGs

**FIGURE 3:**
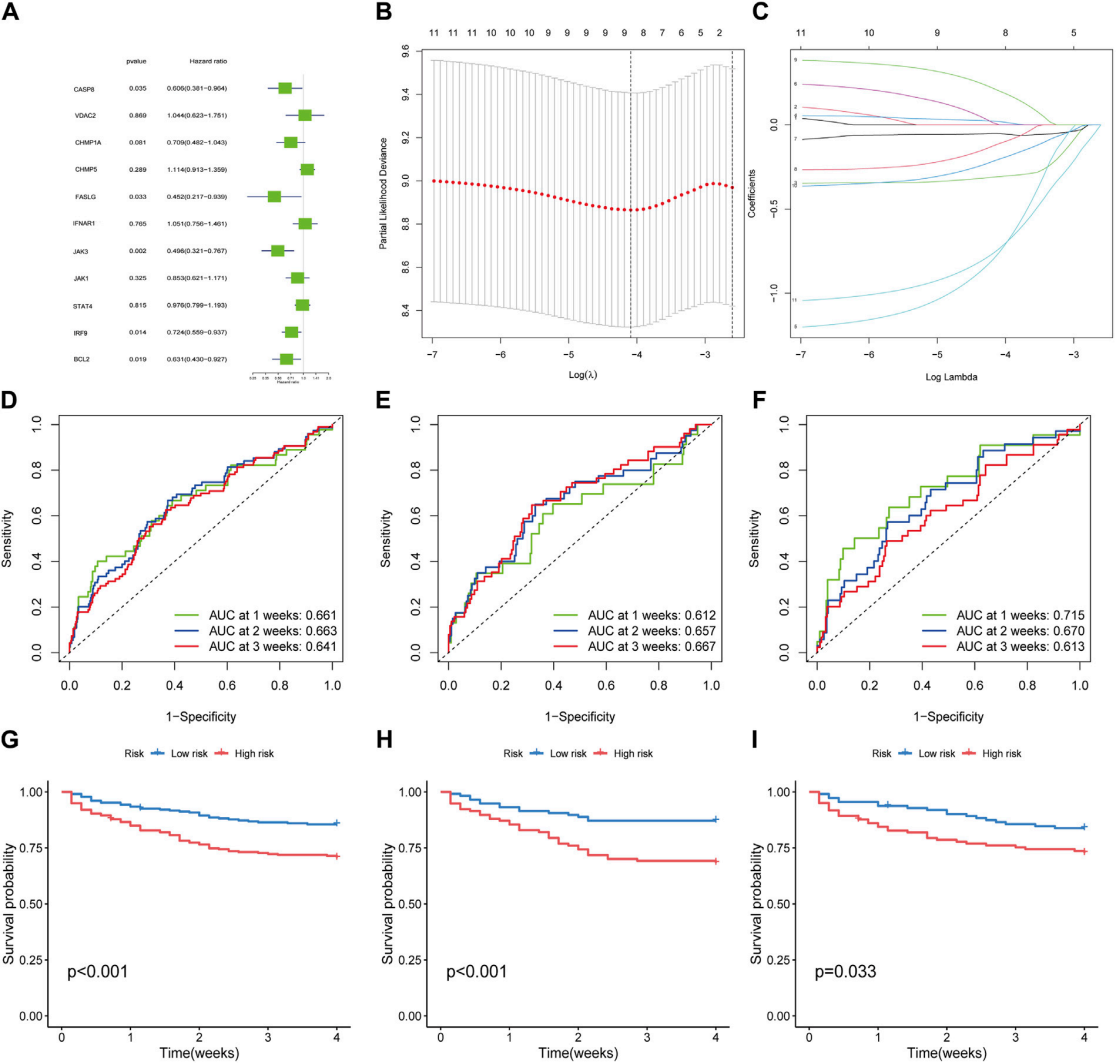
Filtration of hub genes and verification of prognostic value. **(A)** Cox regression model with HR of 11 differentially expressed genes. **(B,C)** Construction of hub gene signature based on LASSO regression. **(D–F)** The time-dependent ROC curves in whole dataset, train dataset and test dataset, respectively. **(G–I)** Kaplan-Meier curves for the overall survival of patients in the high- and low-risk groups in whole, train and test dataset, respectively.

To broaden the clinical significance of DEGs, we integrated survival data from the GSE65682 dataset, then we employed the univariate COX regression (Figure 3A) and LASSO regression (Figures 3B, C) to establish a survival model, ultimately, 4 hub genes: *BCL2, FASLG, IRF9* and *JAK3* were screened out. The AUC for 1, 2, and 3 weeks was calculated in the time-ROC curves (Figures 3D–F). Then, septic patients were stratified into low- and high-risk groups using the median cut-off value based on risk scores and the survival comparison showed that low-risk patients have greater survival time than high-risk ones (Figures 3G–I). The risk survival status charts and expression patterns of four hub genes were presented in Supplementary Figure S2, showing the survival time and rate decreased as the risk scores increased. The algorithms for the time-ROC curve, survival analysis, and expression profiles as described above were run three times separately in the whole, train and test datasets. Additionally, a nomogram model was constructed for overall survival (OS) prediction incorporating *FASLG, JAK3, IRF9, BCL2* and other predictors (gender and age) (Supplementary Figures 3A, D). These results revealed the prognostic capability of hub genes for sepsis, while acknowledging their limitations.

Besides, the GSEA-KEGG analysis indicated that hub genes were closely correlated to certain functional pathways participated in the occurrence of sepsis (Supplementary Figures 3B–E, F). According to GSVA terms (Supplementary Figure S4), we observed that hub genes were enriched for multiple immune response pathways, including antigen processing and presentation, T cell receptor signaling pathway, et al.

### 3.4 Immune landscape analysis

**FIGURE 4:**
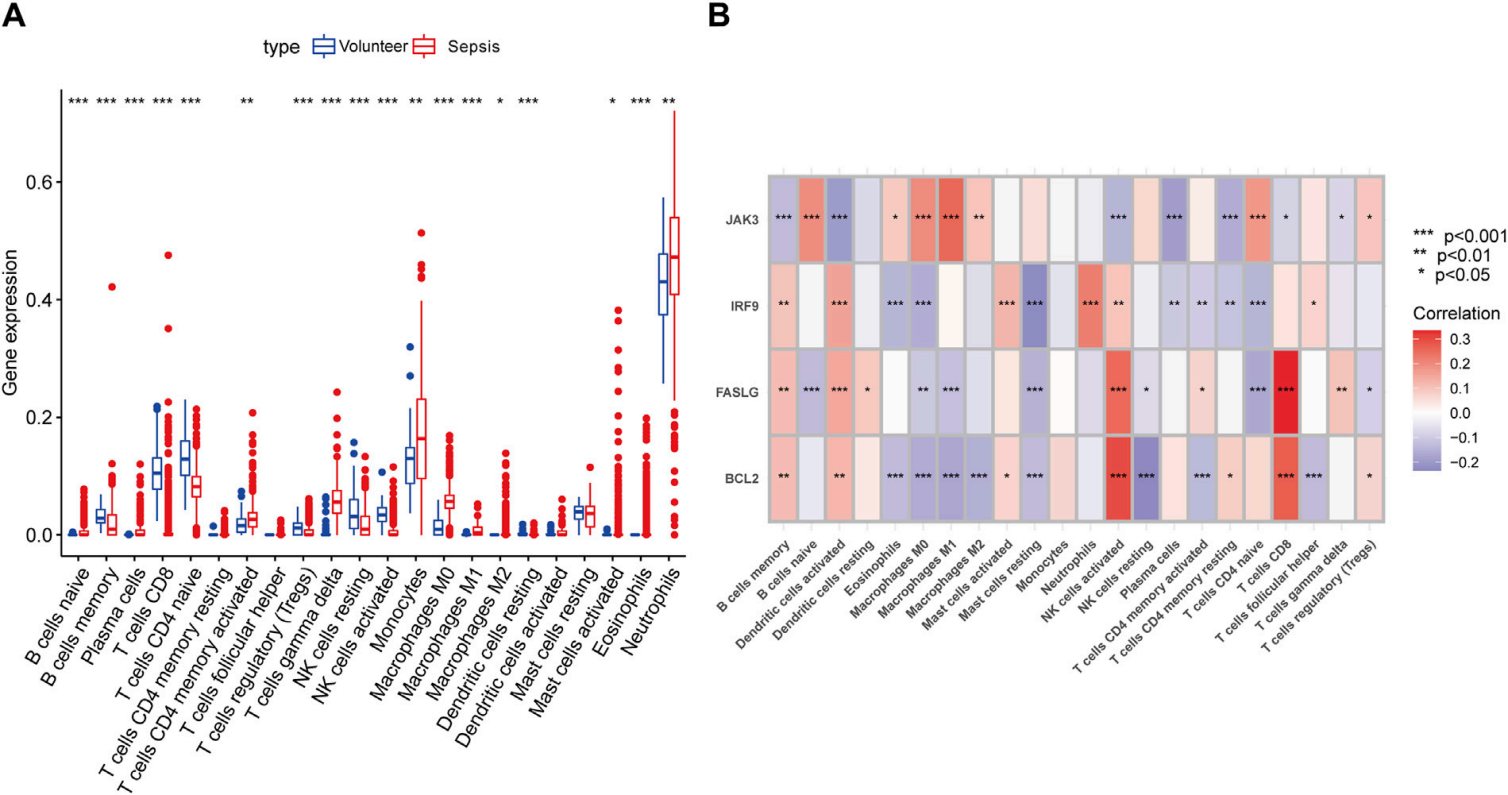
Immune landscape analysis. **(A)** The differences in immune cells between septic patients and healthy volunteers with CIBERSORT algorithm. **(B)** The concrete relationships between hub genes and several immune cells. (*p < 0.05, **p < 0.01, ***p < 0.001).

Previous studies have demonstrated the close connection between sepsis and immune microenvironment (Delano and Ward, 2016; Liu et al., 2022). Therefore, we assessed the concrete changes in immune microenvironment using CIBERSORT (Figure 4A). The proportions of memory B cells and naive CD4^+^ T cells were lower in sepsis than in normal samples, while monocytes, neutrophils, and macrophages were higher in sepsis samples. The results of correlations between hub genes and immune cells showed that NK cells and CD8^+^ T cells had strong positive links with *BCL2* and *FASLG*, *IRF9* was negatively related to CD4^+^ T cells, whereas *JAK3* exhibited a strong positive influence on B cells and CD4^+^ T cells (Figure 4B). Hence, our findings exhibited that apoptosis-related hub genes were inseparable from the changes in immune system in sepsis.

### 3.5 Expression levels of hub genes in scRNA-seq data

**FIGURE 5:**
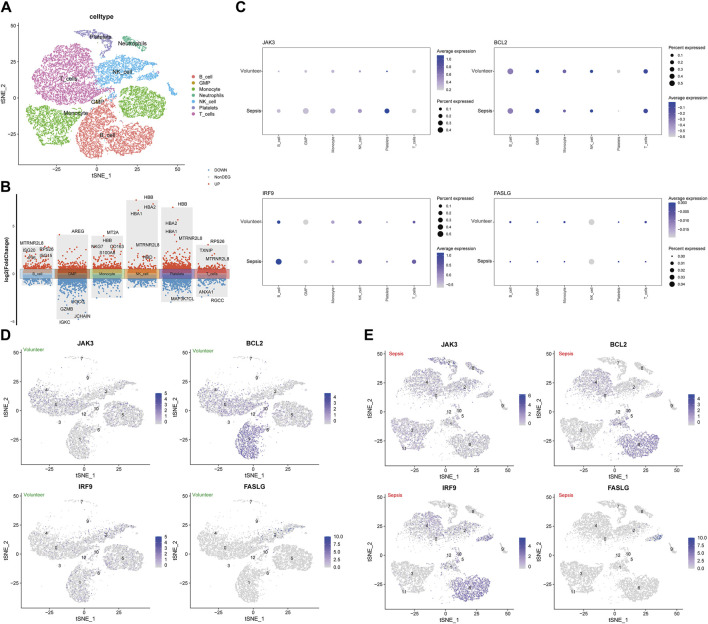
Analysis of single-cell RNA-sequencing data in GSE167363. **(A)** The t-distributed stochastic neighbor embedding (t-SNE) plot. **(B)** The volcano plot of differentially expressed genes in immune cells. **(C)** The associations of four hub genes and immune cells between sepsis group and healthy group. **(D)** The clusters of immune cells for individual hub gene in healthy volunteers. **(E)** The clusters of immune cells for individual hub gene in septic patients.

GSE167363 was downloaded from GEO databases for normalization, scaling, clustering, and highly variable genes screening. The dimensionality-reduced clusters were showed on the 2D map produced with the t-distributed t-SNE (Figure 5A). The clusters predominantly consisted of six immune cell types: T cells, B cells, monocytes, NK cells, platelets and neutrophils, with the former four types accounting for large proportions. Based on the analysis of differentially expressed genes in distinct cell types (Figure 5B), further investigation revealed hub genes showed differential expression across distinct immune cell types. In septic patients, a decrease in the expression levels of *BCL2* was observed in B cells and monocytes compared to healthy samples, the expression levels of *FASLG* in B cells, monocytes, and T cells were lower in septic patients than in healthy subjects. In contrast, higher expression levels of *IRF9* were detected in B cells and T cells, while *JAK3* expression was elevated in B cells and monocytes among septic patients, comparing to healthy volunteers (Figure 5C). Subsequently, hub genes were visually distinguished in cell clusters from healthy cases and sepsis cases using multi-element expression diagrams (Figures 5D, E). These results provided a clearer understanding of the relationships between hub genes and immune cells in sepsis.

### 3.6 Verification of hub genes in external datasets

**FIGURE 6:**
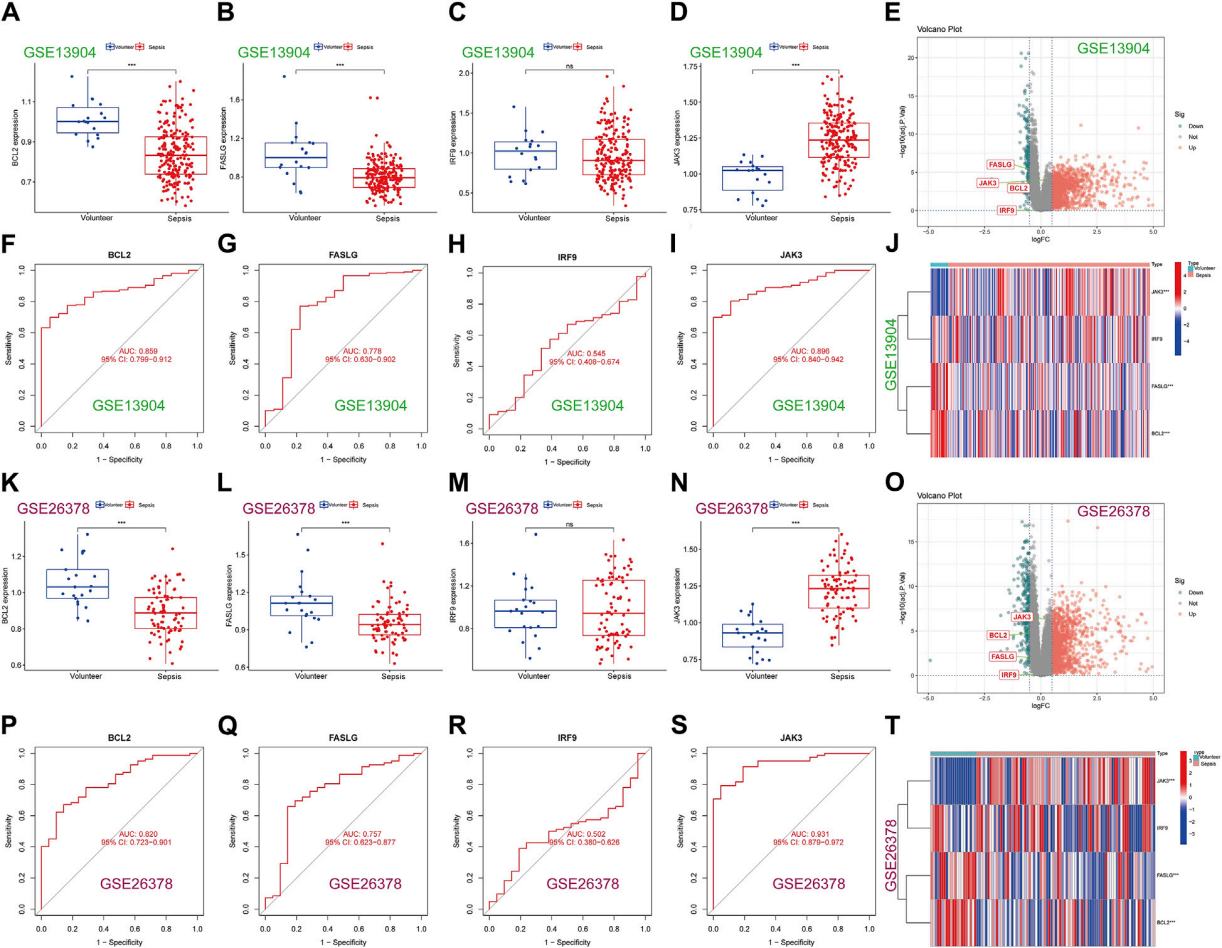
Expression of hub genes in the external validation datasets. **(A–D)** The expression of *BCL2*, *FASLG*, *IRF9* and *JAK3* in GSE13904 dataset, respectively. **(E)** The volcano plot of hub genes in GSE13904 dataset. **(F–I)** The diagnostic ROC curves of *BCL2*, *FASLG*, *IRF9* and *JAK3* in GSE13904 dataset, respectively. **(J)** Heatmap of hub genes in GSE13904 dataset. **(K–N)** The expression of *BCL2*, *FASLG*, *IRF9* and *JAK3* in GSE26378 dataset, respectively. **(O)** The volcano plot of hub genes in GSE26378 dataset. **(P–S)** The diagnostic ROC curves of *BCL2*, *FASLG*, *IRF9* and *JAK3* in GSE26378 dataset, respectively. **(T)** Heatmap of hub genes in GSE26378 dataset. (ns means no significance, *p < 0.05, **p < 0.01, ***p < 0.001).

**FIGURE 7:**
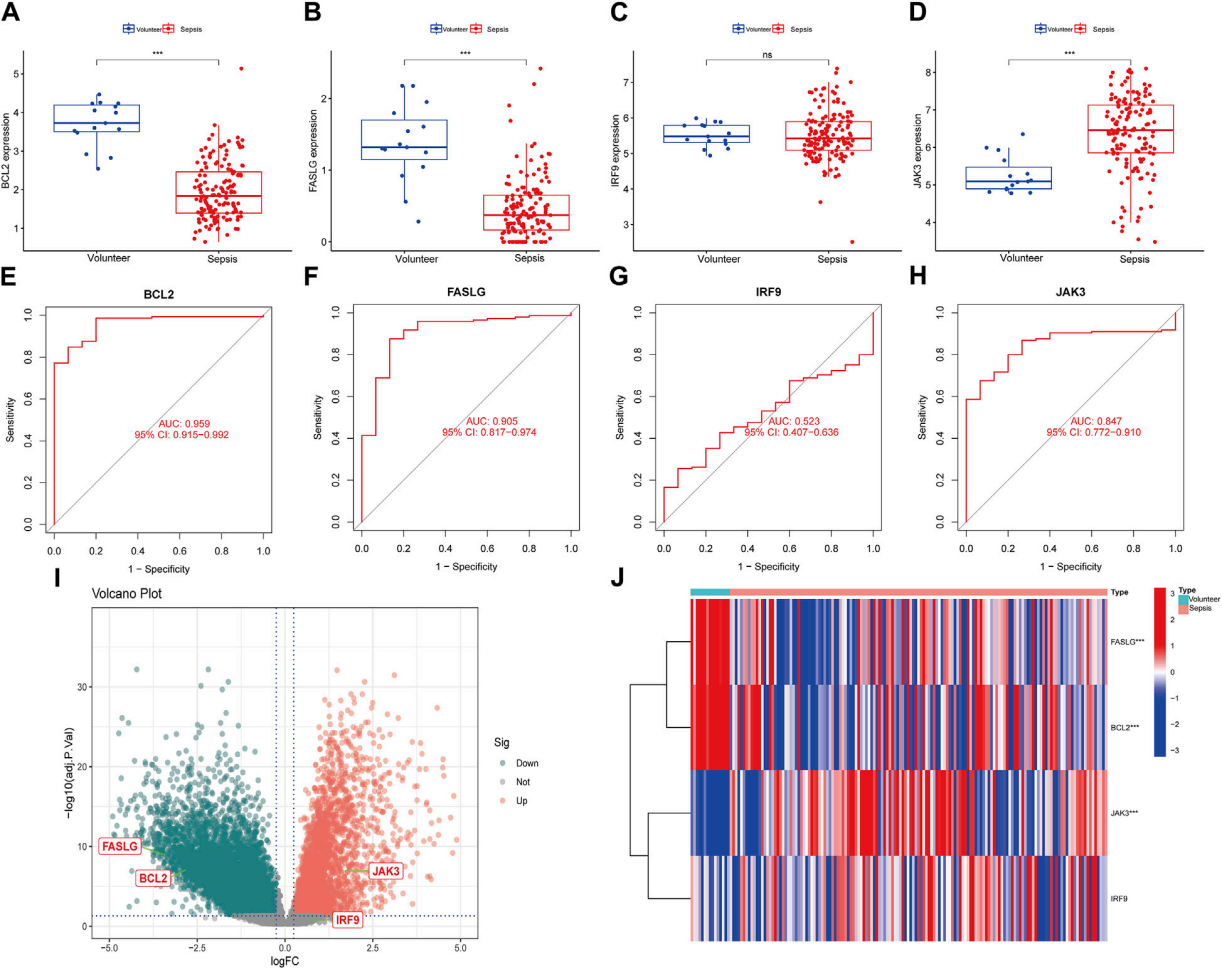
Expression of hub genes in Zhongnan Hospital dataset. **(A–D)** The expression of *BCL2*, *FASLG*, *IRF9* and *JAK3* in Zhongnan Hospital dataset, respectively. **(E–H)** The diagnostic ROC curves of *BCL2*, *FASLG*, *IRF9* and *JAK3* in Zhongnan Hospital dataset, respectively. **(I)** The volcano plot of hub genes in Zhongnan Hospital dataset. **(J)** Heatmap of hub genes in Zhongnan Hospital dataset. (ns means no significance, *p < 0.05, **p < 0.01, ***p < 0.001).

To enhance the reliability of results, the expression levels of *BCL2, FASLG, IRF9* and *JAK3* were investigated in three external datasets. In the GSE13904 dataset (Figures 6A–J), the expression trends of *BCL2* and *FASLG* showed a significant decrease in sepsis, while *JAK3* exhibited elevation. However, there was no significant difference in the expression level of *IRF9* between two groups. The ROC curves for *BCL2*, *FASLG* and *JAK3* demonstrated good AUC values. The volcano plot and heatmap yielded similar results. The verification results of the GSE26378 dataset (Figures 6K–T) and Zhongnan Hospital dataset (Figure 7) closely aligned with the findings from the GSE13904 dataset, confirming the diagnostic advantages of hub genes for septic patients.

On the other hand, we attempted to estimate the prognostic value of individual hub gene. Whereas, the time-ROC curves showed that hub genes did not exhibit prognostic nature in either the Zhongnan Hospital dataset or the GSE65682 dataset (Supplementary Figure S5).

### 3.7 RT-qPCR and IHC validation of the hub genes

**FIGURE 8:**
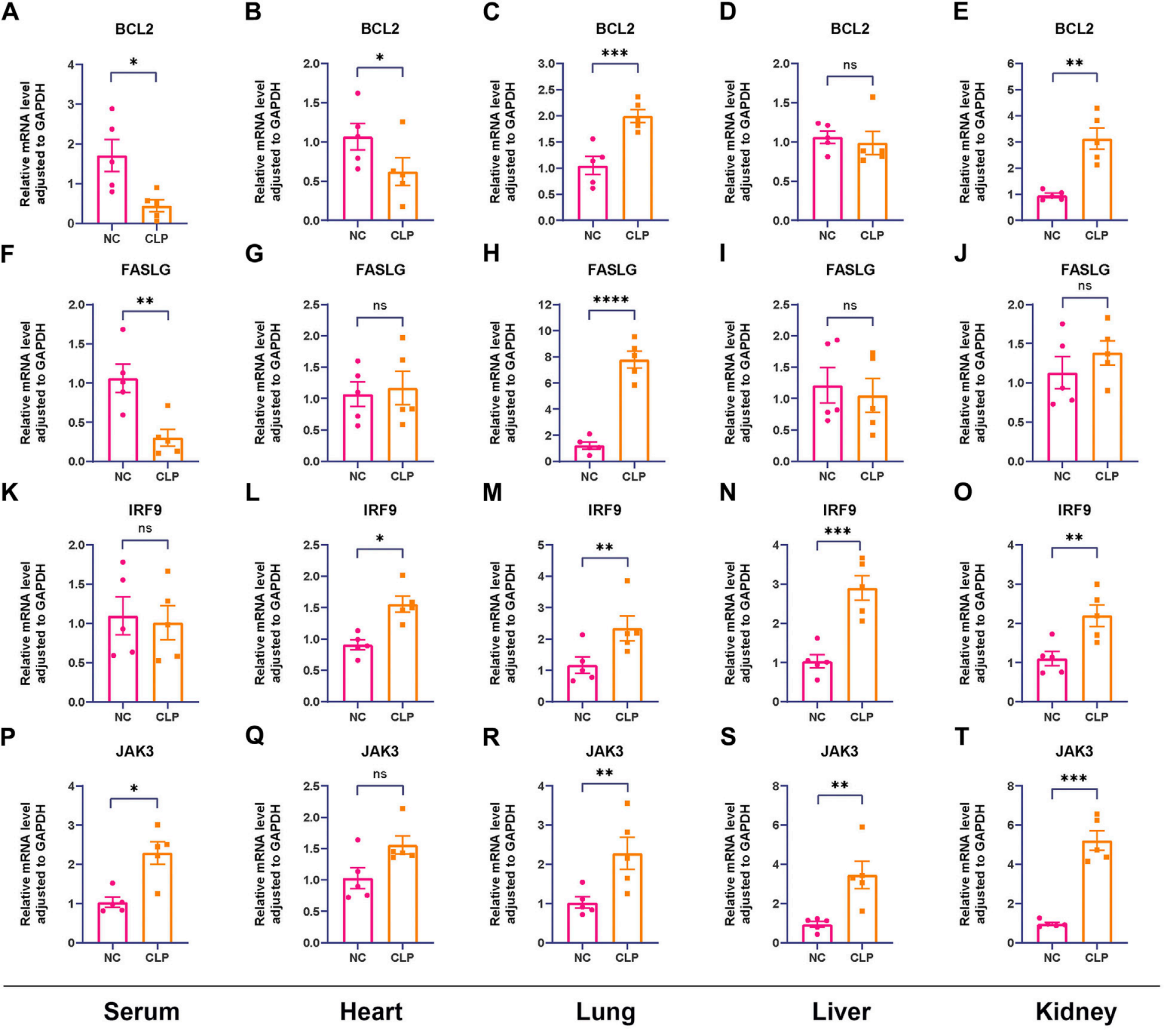
Confirmation of relative expression levels of hub genes between control group and CLP group in animal model using RT-qPCR (n = 5). The analyses were performed in serum samples, heart tissues, lung tissues, liver tissues and kidney tissues, respectively. **(A–E)** The expression levels of *BCL2* in serum, heart, lung, liver and kidney. **(F–J)** The expression levels of *FASLG* in serum, heart, lung, liver and kidney. **(K–O)** The expression levels of *IRF9* in serum, heart, lung, liver and kidney. **(P–T)** The expression levels of *JAK3* in serum, heart, lung, liver and kidney. (ns means no significance, *p < 0.05, **p < 0.01, ***p < 0.001).

**FIGURE 9:**
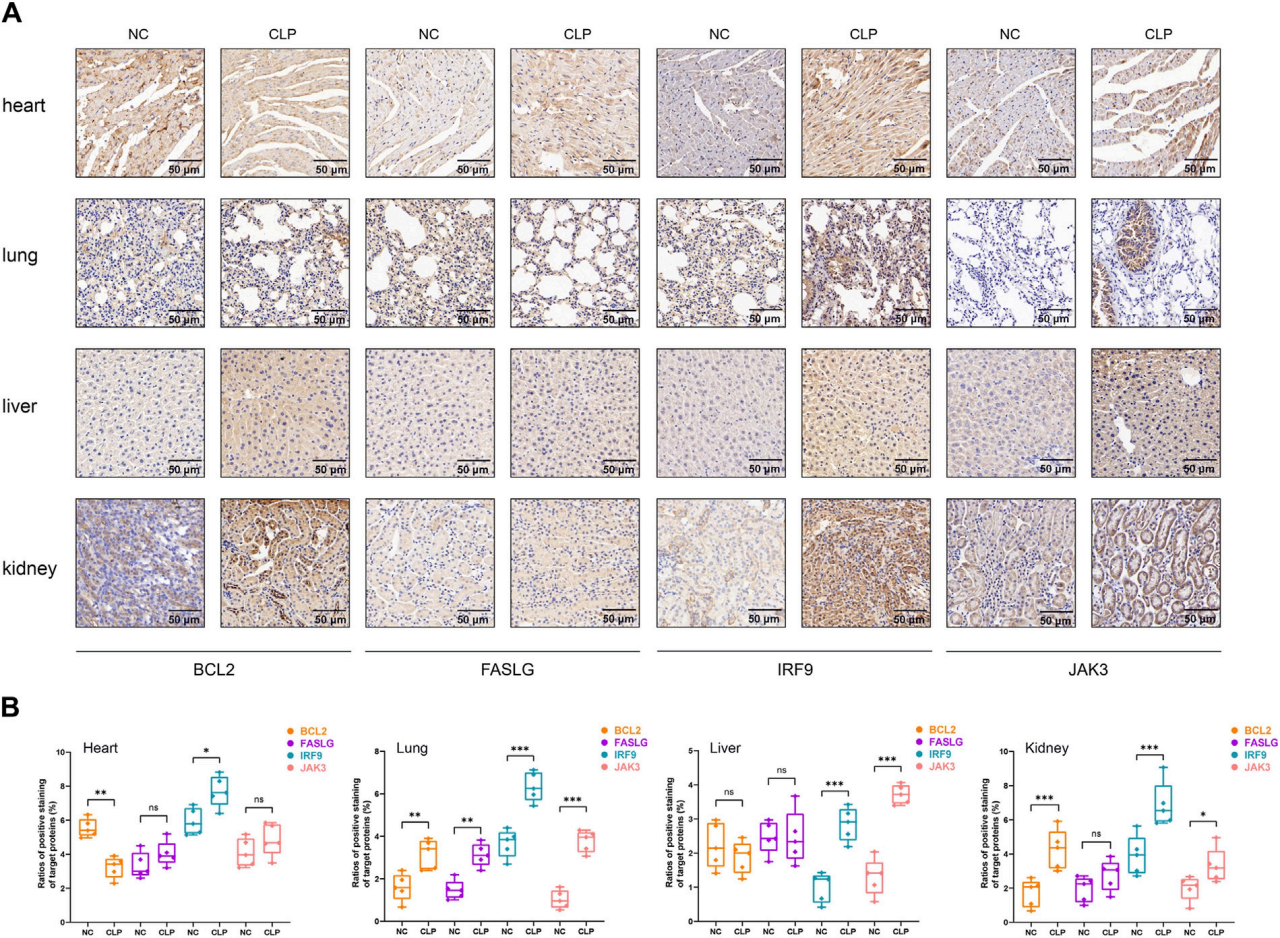
The IHC analysis of hub genes. **(A)** Representative IHC staining of *BCL2*, *FASLG*, *IRF9* and *JAK3* in four organs at 30* magnification. The target proteins were stained in brown. Scale bar = 50 µm. **(B)** Quantitative analysis of positive rate of hub genes in heart, lung, live and kidney, respectively. In statistical analysis, ns means no significance, *p < 0.05, **p < 0.01, ***p < 0.001.

To start with, the serum levels of Cr and BUN of sepsis group were significantly higher than that of control group (Supplementary Figure S6). Subsequently, the serum levels of hub genes were depicted in Figures 8A, F, K, P, in which we observed elevated levels of *JAK3* and decreased levels of *BCL2* and *FASLG* in sepsis group compared to controls, providing further compelling evidence corresponding to the results from external datasets. Considering the general impact of sepsis on various organs, the expressions of hub genes were assessed in four major organs. In the heart (Figures 8B, G, L, Q), *IRF9* expression was upregulated in sepsis, while *BCL2* was downregulated. However, there was no intergroup difference in the expression levels of *FASLG* and *JAK3*. In the lung (Figures 8C, H, M, R), all four hub genes exhibited significant upregulation in the CLP group. In the liver (Figures 8D, I, N, S), *IRF9* and *JAK3* were elevated in the sepsis model, while no difference was observed in the expression of *BCL2* and *FASLG* between two groups. In the kidney (Figures 8E, J, O, T), *BCL2*, *IRF9*, and *JAK3* displayed upregulation in the CLP group. The IHC results manifested the representative positive expression of hub genes as brown staining (Figure 9A). The quantification of IHC results (Figure 9B) corroborated the differences in hub gene expression between two groups, consistent with the RT-qPCR analysis. Besides, the TUNEL assay was performed on the four organs as routine detection for apoptosis (Supplementary Figure S7), in which the apoptotic cells significantly increased in the sepsis group.

## 4 Discussion

Sepsis keeps a critical clinical issue and many efforts have been made to improving its management. The pathophysiological mechanisms of sepsis are complicated, however, studies focused on cell death have expanded a novel perspective. In our report, the acknowledged public datasets and machine learning were used to determine the role of apoptosis in sepsis and identify 11 DEGs including: *CASP8, VDAC2, CHMP1A, CHMP5, FASLG, IFNAR1, JAK1, JAK3, STAT4, IRF9* and *BCL2*. Further, 4 hub genes (*BCL2, FASLG, JAK3* and *IFR9*) were emerged as remarkable diagnostic signatures, which was supported by validation of external datasets. Based on immune infiltration analysis and scRNA-seq data, we verified the underlying associations between hub genes and immune cells. Additionally, the differential expression levels of hub genes in separate organs provided more indications for their potential impacts on sepsis-induced organ injury.

To date, molecular biology has broadened the function recognition of identified hub genes. *BCL2*, known as B-cell leukemia/lymphoma gene number 2, manipulates cell survival to deploy typical anti-apoptotic effects in normal cellular lineages (Ploner et al., 2008; Kang et al., 2011). It has been demonstrated that the expression of *BCL2* decreased in white blood cells in septic patients compared to control subjects (Lorente et al., 2021), which was aligned with our findings. By contrast, we paid much attention to the role of *BCL2* in non-apoptotic pathways. Researchers reported that necroptosis pathway (another form of PCD) was significantly activated in sepsis (Shashaty et al., 2019; Reilly et al., 2022). Han She et al. (She et al., 2023) identified *BCL2* as one of the necroptosis-related hub genes in sepsis, highlighting its diagnostic and prognostic value. Moreover, necroptosis and pyroptosis (another form of PCD) pathways collaborated to aggravate tissue injury in the process of sepsis (Chen et al., 2020), and *BCL2* constrained the induction of these two pathways through interaction with a BCL2-homology-3 like domain (Shi and Kehrl, 2019). Hence, the effects of *BCL2* are not limited to apoptosis, but also encompass other forms of PCD, which may be attributed to the function of *BCL2* as the molecular death switch and partially overlapping signaling transduction pathways in PCD (Borkan, 2016). The exact role of *BCL2* in the crosstalk among different cell death pathways remains unclear, requiring deeper validation in the future. Additionally, *BCL2* was proved to produce almost complete protection against T cell apoptosis in transgenic mice that overexpress *BCL2* (Hotchkiss et al., 1999; Hotchkiss et al., 2000; Wesche-Soldato et al., 2007a). The naive T cells, naive B cells and NK cells were dependent on individual *BCL2* molecule (Carrington et al., 2017). More importantly, a cohort study in pediatric intensive care unit (PICU) showed patients, who developed sepsis and/or multiple organs dysfunctions (MODS), had lower lymphocytic counts and lower levels of *BCL2* comparing to control group (El Shazly et al., 2018), which provided additional proof covering different age groups. Furthermore, in our study, *BCL2* was positively correlated with B cells, T cells while negatively correlated to NK cells, meanwhile, *BCL2* was related to several immune-related signaling pathways including antigen processing and presentation, the T cell receptor signaling pathway, and ECM receptor interaction (Sutherland et al., 2023). Hence, our research provides innovative insights into how apoptosis interacted with host immune response in sepsis.

*FASLG* (Fas ligand) is a tumor necrosis factor (TNF), binding to FAS receptor to initiate an extrinsic apoptosis pathway (Audo et al., 2014). In a prospective cohort enrolled septic patients (Yoo et al., 2021), there was a significant difference in 90-day mortality between low and high serum concentrations of *FASLG*, further, we provided the novel results of diagnostic value of *FASLG* for sepsis. According to Soldato’s report (Wesche-Soldato et al., 2007b), CD8^+^ T cells expressed *FASLG* were detrimental to liver injury after CLP. Our results demonstrated strong correlations between *FASLG* expression and CD8^+^ T cells, CD4^+^ T cells and memory B cells. Thus, it is highly likely that *FASLG* is involved in the regulation of immune response in sepsis.

The interferon regulatory factors (IRFs) are identified to be a family of transcription factors which play crucial roles in immune response (Jefferies, 2019). Most family members participate in the production of type I interferons and regulation of undifferentiated immune cell development. (Honda et al., 2006; Tamura et al., 2008). Unlike these members, *IRF9* interact with phosphorylated STAT1 and STAT2 dimer to form interferon-stimulated gene factor 3 (ISGF3), the transcriptionally active complex (Qureshi et al., 1995). In our study, *IRF9* was filtered as one of the hub genes based on machine learning, however, the noteworthy issue is the absence of differential expression of *IRF9* between healthy volunteers and septic patients in external datasets confirmation. Several possible reasons were analyzed as following (Kumar, 2018): It is difficult to detect the independent expression of *IRF9* in sepsis because its main function is to assemble trimolecular ISGF3 complex with phosphorylated STAT1 and STAT2, which then translocates to the nucleus (Fu et al., 1990). According to Lau’s study (Lau et al., 2000), dimerization is required to retain *IRF9* in the cytoplasm and *IRF9* tend to pre-associate with STAT2 in the non-stimulated state (Hawiger et al., 2015); Considering the complicated information conveyed in gene expression datasets, *IRF9* is constitutively expressed in most, instead of all human and murine tissues (Suprunenko and Hofer, 2016; Stone, 2017) There was defective interferon antiviral responses in both adult and pre-school children in asthma model, serving as valuable inspiration for sepsis (Bergauer et al., 2017). Consequently, in-depth research is still required to validate these assumptions and disclose the underlying molecular mechanism of regulation of *IRF9* on sepsis.

Janus Kinase (JAK) is a family of non-receptor tyrosine kinases which participate in JAK-STAT pathway including *JAK3* (Bousoik and Montazeri Aliabadi, 2018; Agashe et al., 2022). During eryptosis (suicidal programmed death of mature red blood cells) (Jemaa et al., 2017), *JAK3* was activated by energy depletion, subsequently stimulated eryptosis in turn, which was blunted by pharmacologic inhibitors or genetic knockout of *JAK3* (Bhavsar et al., 2011). Long et al. (Long et al., 2022) investigated the correlation between *JAK3* expression and tumor microenvironment immune cell infiltration, proved elevated *JAK3* expression linked to higher infiltration of immune cells. In their data, *JAK3* expression was positively correlated with B cells, CD4^+^ T cells, CD8^+^ T cells, and dendritic cells. In sepsis, we have identified *JAK3* as an apoptosis-related hub gene for sepsis and explored its participation in immune regulation, in order to provide a better picture of the nature of these pathological interactions.

More importantly, the behavior of hub genes appeared not homogeneous in different organs in mouse model. Previous publications reported that *BCL2* was upregulated in the kidney tissue in LPS-induced sepsis while downregulated in the heart in ischemia-reperfusion model (Ren et al., 2020; Xia et al., 2023). Additionally, lung was regarded as a specific organ where *FASLG* was constitutively expressed. In response to viral infection, the intense inflammatory response allowed the upregulation of *FASLG* expression in lung tissues (Koshkina et al., 2020; Li et al., 2020). *IRF9* was upregulated in the heart tissue and modulated the cardiomyocyte death and inflammation development after myocardial ischemia reperfusion (Zhang et al., 2014). In autoimmune hepatitis, the expression of *JAK3* was found to be increased, affecting inflammatory cytokine production (Asselah et al., 2003; Centa et al., 2023). In the context of sepsis, our findings yielded partially similar expression trends of hub genes in target organs, while presenting novel discoveries compared to above literature. Our analysis indicated the apoptosis-related genes played distinct roles in the sepsis-induced multiple organ damage. More investigations are warranted to unveil the molecular mechanism of regulation of hub genes on sepsis.

However, our study has some limitations. First, we did not explore the explicit molecular mechanisms of how apoptosis-related genes regulate sepsis. Second, our study did not account for the added value of hub genes and other diagnostic features of sepsis. Third, considering the validation datasets covering different age groups, the relevant results could be affected by biological differences among different populations. Fourth, the heterogeneity of expression patterns of hub genes across various organs requires in-depth research in the future.

## 5 Conclusion

In summary, we systematically identified 11 apoptosis-related differentially expressed genes in sepsis, and four hub genes (*BCL2*, *FASLG*, *JAK3* and *IRF9*) were recognized as valuable diagnostic biomarkers. Furthermore, we explored the correlations from hub genes to the immune microenvironment of sepsis. For the first time, we revealed the relationship between apoptosis pathway and sepsis from bioinformatics perspective, constituting a reference for basic research and clinical decision-making.

## Data Availability

The datasets presented in this study can be found in online repositories. The names of the repository/repositories and accession number(s) can be found in the article/Supplementary Material.
