# Supplementary material for: Identification of apoptosis-immune-related gene signature and construction of diagnostic model for sepsis based on single-cell sequencing and bulk transcriptome analysis
Source: Front Genet. 2024 Jun 4;15:1389630. doi: 10.3389/fgene.2024.1389630 (PMC11183325; doi:10.3389/fgene.2024.1389630)
Supplement: Supplementary file 1 [file Table1.docx]

**Supplementary Table 1. Demographic and clinical characteristics of recruited samples in Zhongnan Hospital: 143 septic patients (case) and 15 healthy volunteers (control).**

| **Characteristics** | **Samples, No. (%)** | |
| --- | --- | --- |
|  | **Case^a^ (n=143)** | **Control^b^ (n=15)** |
| Sex | | |
| Male | 98 (68.5%) | 13 (86.7%) |
| Female | 45 (31.5%) | 2 (13.3%) |
| Age, years | | |
| Median years | 52 years | 29 years |
| 18-30 y | 8 (5.6%) | 14 (93.3%) |
| 31-49 y | 38 (26.6%) | 1 (6.7%) |
| 50-80 y | 97 (67.8%) | 0 (0%) |
| Charlson Comorbidity Index | | |
| 0 | 5 (3.5%) | 15 (100%) |
| 1 | 97 (67.8%) | 0 (0%) |
| 2 | 31 (21.7%) | 0 (0%) |
| ≥3 | 10 (7.0%) | 0 (0%) |

^a^Inclusion criteria for septic patients: Patients were diagnosed as sepsis or septic shock according to ‘Sepsis-3’ consensus definition, aged between 18-80 years, without drug allergies, hospitalization records were complete.

^b^Inclusion criteria for healthy volunteers: The general health of volunteers was good, aged 18-80 years, without drug allergies, volunteers denied previous medical history of hypertension, diabetes mellitus, coronary artery disease or stroke, etc.


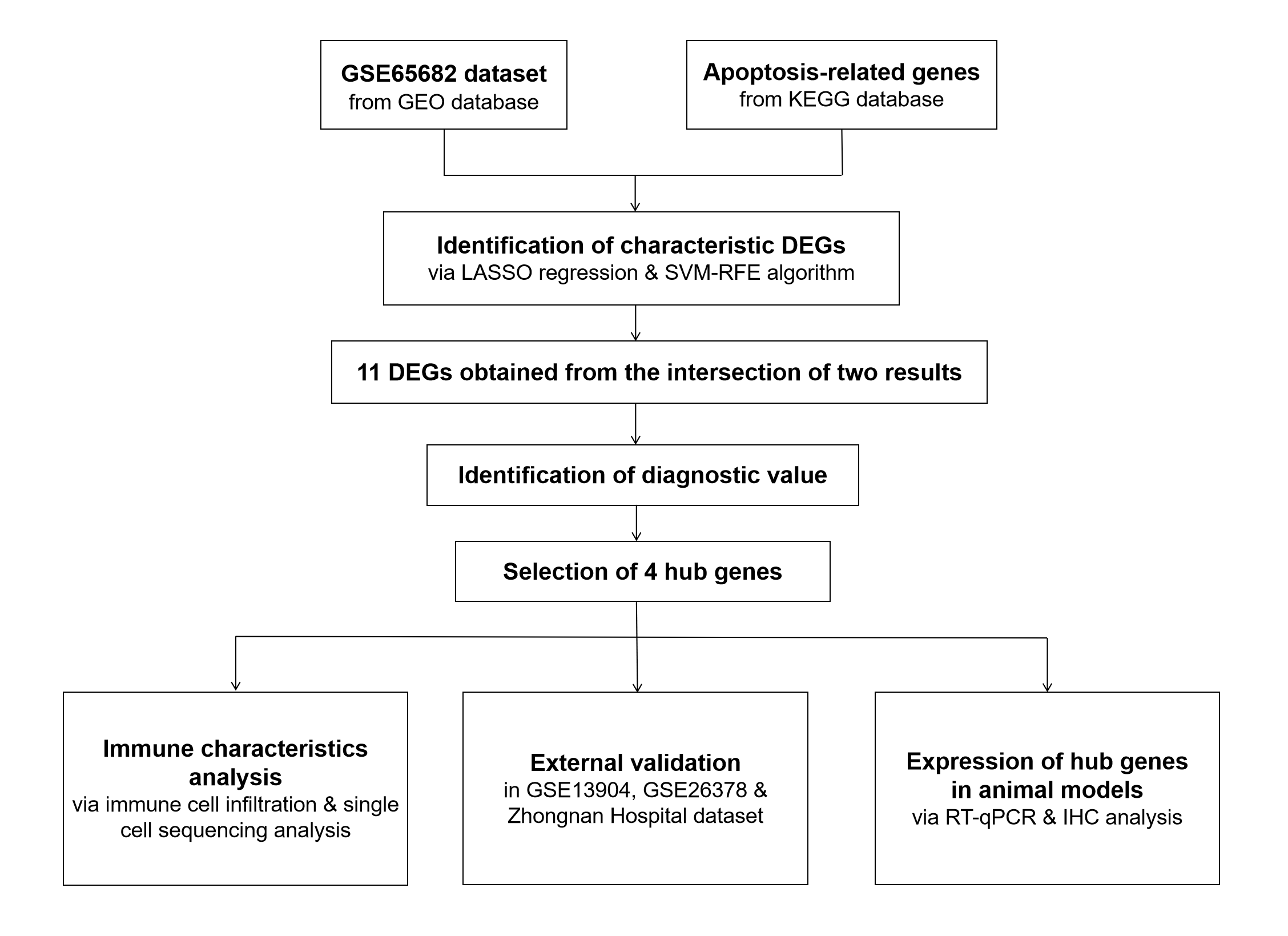


**Supplementary Figure 1.** The main research process of the study.


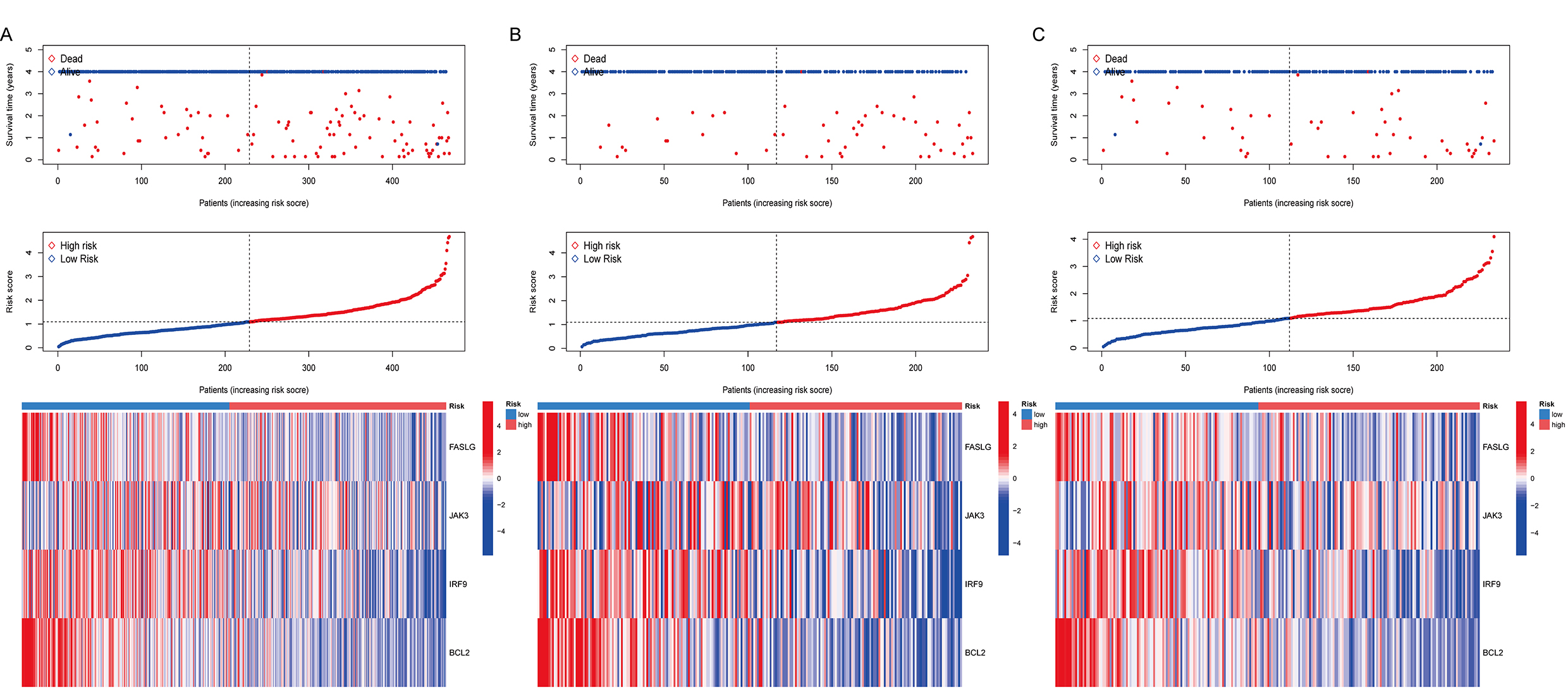


**Supplementary Figure 2.** Verification of prognostic values of four hub genes based on GSE65682. Survival overview, risk score distribution and expression heatmap of hub genes in (A) whole dataset, (B) train dataset, and (C) test dataset.


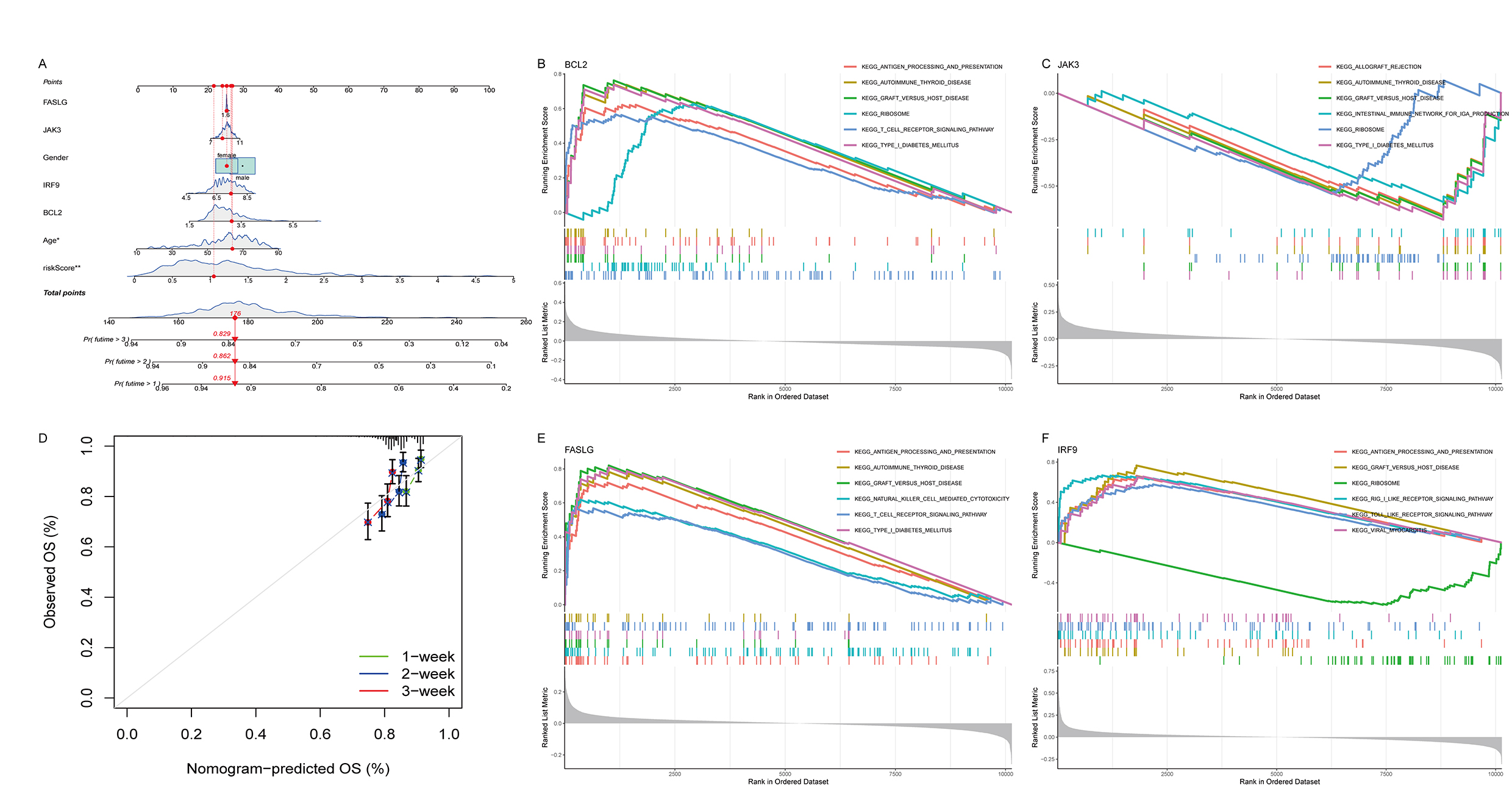


**Supplementary Figure 3.** Nomogram development and GSEA analysis of hub genes. (A) Each variable value points to ‘Points’ bar and total points are used to calculate the 1-, 2- and 3-week survival probability. (D) The calibration curves. (B) Single gene GSEA-KEGG pathway analysis of *BCL2*. (C) Single gene GSEA-KEGG pathway analysis of *JAK3*. (E) Single gene GSEA-KEGG pathway analysis of *FASLG*. (F) Single gene GSEA-KEGG pathway analysis of *IRF9*.


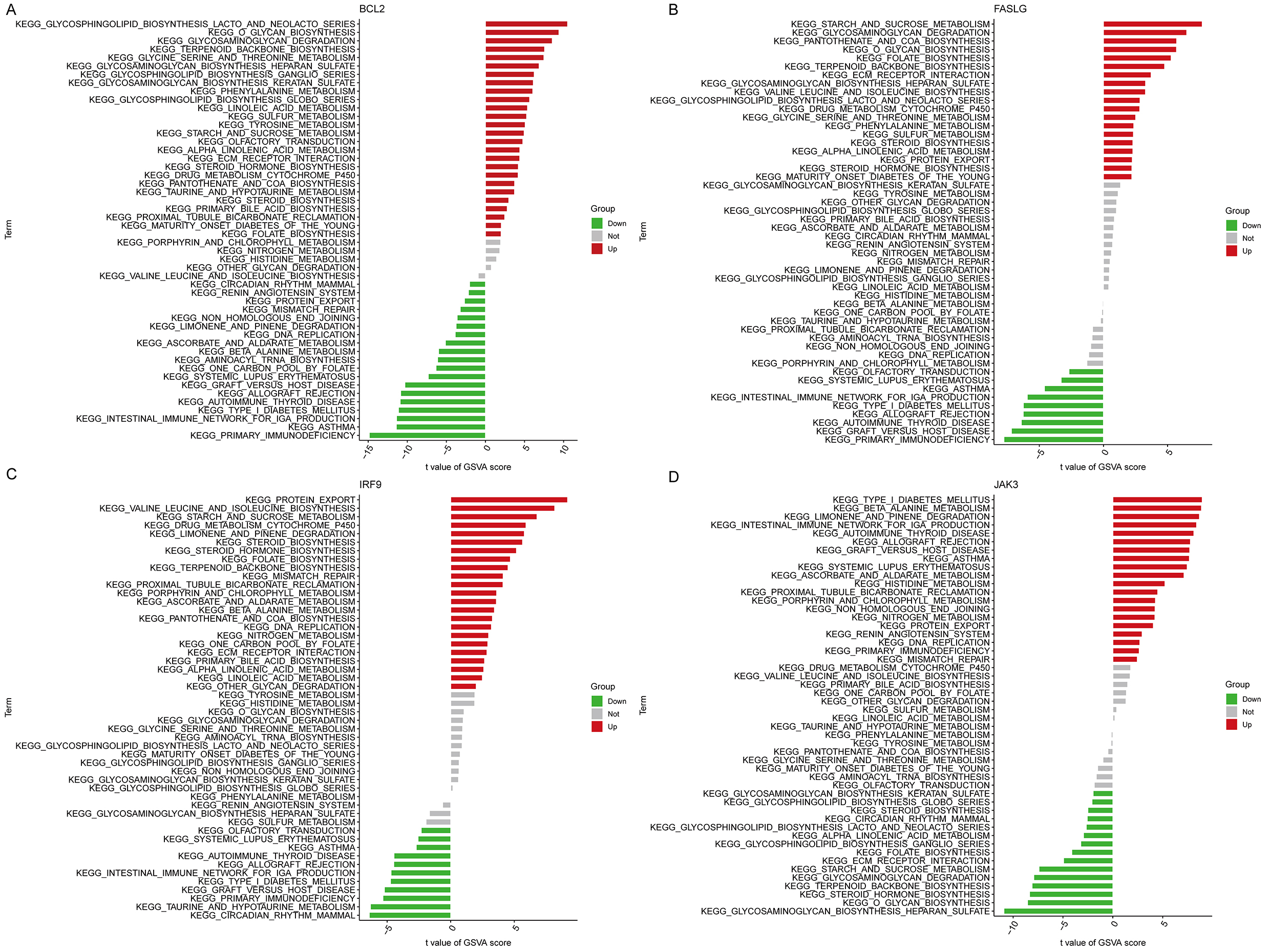


**Supplementary Figure 4.** Up- and down- regulated GSVA terms based on each hub gene. (A) GSVA terms linked to *BCL2*. (B) GSVA terms linked to *FASLG*. (C) GSVA terms linked to *IRF9*. (D) GSVA terms linked to *JAK3*.


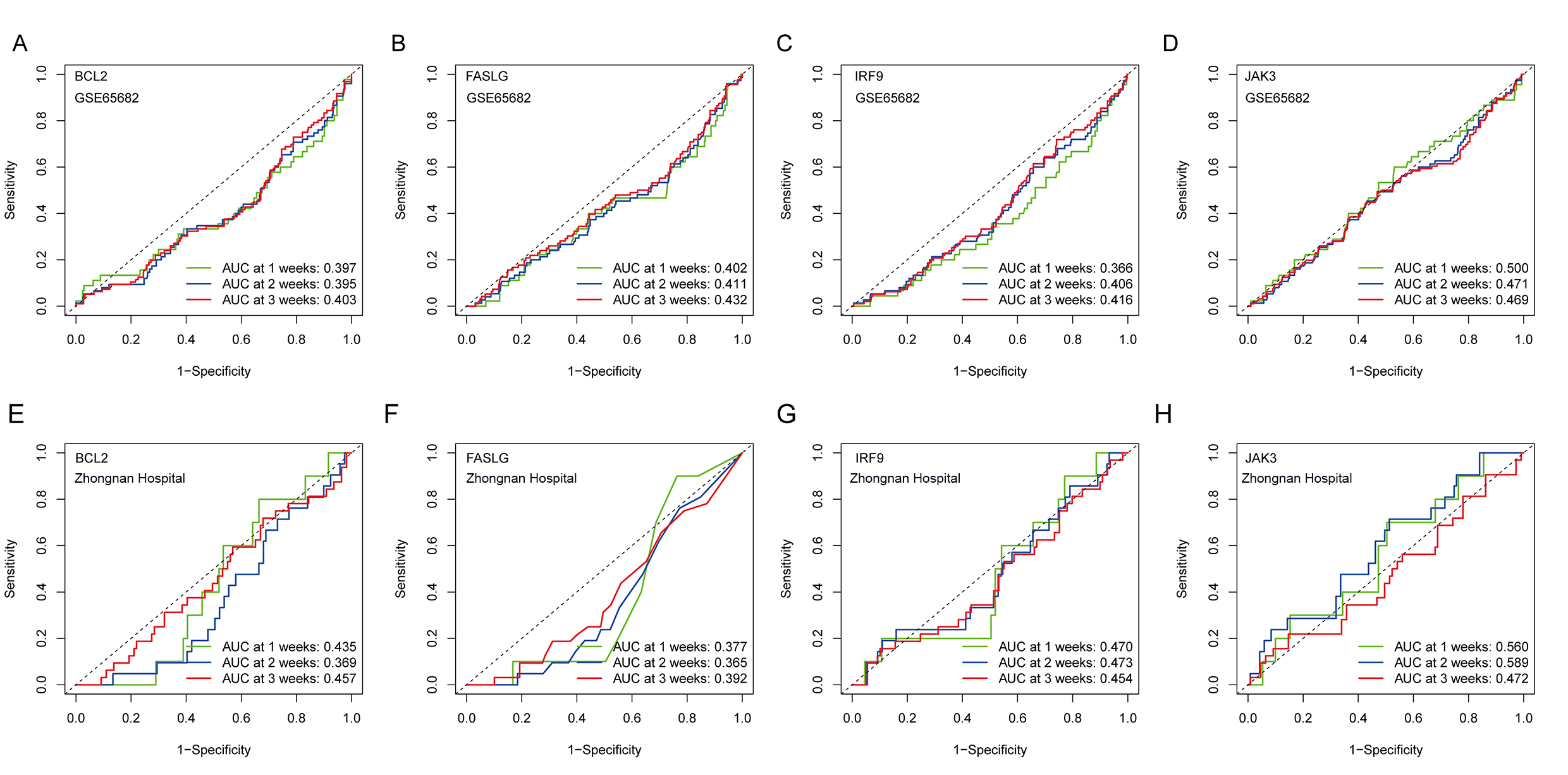


**Supplementary Figure 5.** The time-dependent ROC curves of individual hub gene. (A) The ROC curves of *BCL2* in GSE65682 dataset. (B) The ROC curves of *FASLG* in GSE65682 dataset. (C) The ROC curves of *IRF9* in GSE65682 dataset. (D) The ROC curves of *JAK3* in GSE65682 dataset. (E) The ROC curves of *BCL2* in Zhongnan Hospital dataset. (F) The ROC curves of *FASLG* in Zhongnan Hospital dataset. (G) The ROC curves of *IRF9* in Zhongnan Hospital dataset. (H) The ROC curves of *JAK3* in Zhongnan Hospital dataset.


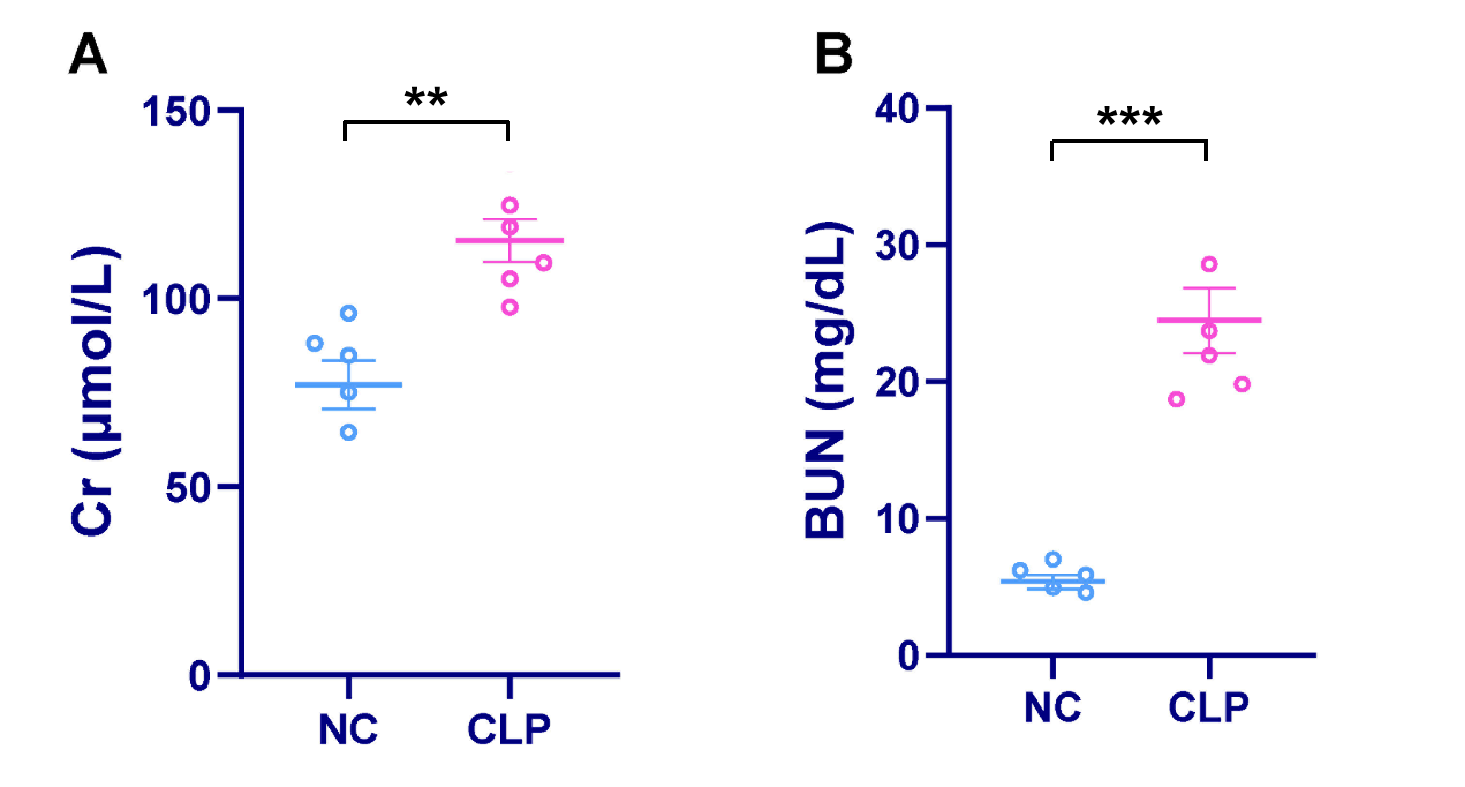


**Supplementary Figure 6.** The creatinine (Cr) levels and blood urea nitrogen (BUN) levels in control and sepsis groups (n=5).





**Supplementary Figure 7.** The TUNEL analysis. (A) The paraffin sections of heart, lung, liver and kidney were stained with TUNEL as described in methods. The representative images were presented at 30* magnification with scale bar=50 µm. (B) Statistical analysis of the rate of TUNEL-positive cells in four organs (**p* < 0.05, ***p* < 0.01, ****p* < 0.001).
